# Development of a diagnostic model for detecting mild cognitive impairment in young and middle-aged patients with obstructive sleep apnea: a prospective observational study

**DOI:** 10.3389/fneur.2024.1431127

**Published:** 2024-08-21

**Authors:** Shuo Wang, Ji-min Fan, Mian-mian Xie, Jiao-hong Yang, Yi-ming Zeng

**Affiliations:** ^1^The School of Nursing, Fujian Medical University, Fuzhou, China; ^2^Department of Respiratory Pulmonary and Critical Care Medicine, The Second Affiliated Hospital of Fujian Medical University, Quanzhou, China; ^3^Respirology Medicine Center of Fujian Province, Quanzhou, China; ^4^The Sleep Medicine Key Laboratory of Fujian Province Universities, Quanzhou, China; ^5^Fujian Provincial Key Laboratory of Lung Stem Cells, The Second Affiliated Hospital of Fujian Medical University, Quanzhou, China; ^6^Jinan Microecological Biomedicine Shandong Laboratory, Jinan, China

**Keywords:** obstructive sleep apnea, mild cognitive impairment, clinical prediction model, diagnostic model, nomogram

## Abstract

**Objectives:**

Obstructive sleep apnea (OSA) is a common sleep-disordered breathing condition linked to the accelerated onset of mild cognitive impairment (MCI). However, the prevalence of undiagnosed MCI among OSA patients is high and attributable to the complexity and specialized nature of MCI diagnosis. Timely identification and intervention for MCI can potentially prevent or delay the onset of dementia. This study aimed to develop screening models for MCI in OSA patients that will be suitable for healthcare professionals in diverse settings and can be effectively utilized without specialized neurological training.

**Methods:**

A prospective observational study was conducted at a specialized sleep medicine center from April 2021 to September 2022. Three hundred and fifty consecutive patients (age: 18–60 years) suspected OSA, underwent the Montreal Cognitive Assessment (MoCA) and polysomnography overnight. Demographic and clinical data, including polysomnographic sleep parameters and additional cognitive function assessments were collected from OSA patients. The data were divided into training (70%) and validation (30%) sets, and predictors of MCI were identified using univariate and multivariate logistic regression analyses. Models were evaluated for predictive accuracy and calibration, with nomograms for application.

**Results:**

Two hundred and thirty-three patients with newly diagnosed OSA were enrolled. The proportion of patients with MCI was 38.2%. Three diagnostic models, each with an accompanying nomogram, were developed. Model 1 utilized body mass index (BMI) and years of education as predictors. Model 2 incorporated N1 and the score of backward task of the digital span test (DST_B) into the base of Model 1. Model 3 expanded upon Model 1 by including the total score of digital span test (DST). Each of these models exhibited robust discriminatory power and calibration. The C-statistics for Model 1, 2, and 3 were 0.803 [95% confidence interval (CI): 0.735–0.872], 0.849 (95% CI: 0.788–0.910), and 0.83 (95% CI: 0.763–0.896), respectively.

**Conclusion:**

Three straightforward diagnostic models, each requiring only two to four easily accessible parameters, were developed that demonstrated high efficacy. These models offer a convenient diagnostic tool for healthcare professionals in diverse healthcare settings, facilitating timely and necessary further evaluation and intervention for OSA patients at an increased risk of MCI.

## 1 Introduction

Obstructive sleep apnea (OSA) is a highly prevalent disorder characterized by impaired breathing that can occur regardless of hypoxia, hypercapnia, or fragmented sleep (1). Globally, OSA impacts nearly a billion individuals, with prevalence rates exceeding 50% in some nations (2). The presence of disordered breathing during sleep has been observed to precipitate the onset of Mild cognitive impairment (MCI) by approximately 10 years (3). MCI is recognized as a prodromal condition for dementia (4). In contrast to the irreversible pathophysiological alterations characteristic of dementia, timely identification and intervention for MCI can potentially prevent or delay the onset of dementia (5). Effective management of OSA through the application of continuous positive airway pressure (CPAP) has demonstrated significant improvements in global cognitive functioning, executive functioning, attention, and memory (6, 7). Notably, individuals diagnosed with both OSA and mild cognitive impairment (OSA + MCI) often display a diminished recognition of their cognitive impairments compared to those without OSA (8). Therefore, early diagnosis of MCI in patients with OSA is extremely necessary.

The Montreal Cognitive Assessment (MoCA) is a highly utilized screening tool, known for its high sensitivity and specificity in detecting MCI (9). Although the MoCA tool is comprehensive, it requires over 10 min to complete and involves 11 distinct tasks, each with its own set of instructions. Therefore, its complexity and the need for specialized training to administer it effectively limits its use in various healthcare settings, particularly in healthcare settings where experienced neurologists are scarce. Consequently, there is a pressing need for the development of convenient and universally applicable tools for MCI screening that can be utilized across different healthcare environments.

A recent study introduced a multimodal predictive model for assessing cognitive impairment risk in OSA patients (10). As the model required the urine biomarker AD7c-NTP, its clinical application may be challenging and economically infeasible. Furthermore, a recent study devised a predictive model for MCI in OSA patients aged ≥45 years (11). The predictive model’s reliance on a lengthy self-compiled lifestyle scale for one of its factors renders it less straightforward and convenient. Moreover, OSA is a heterogeneous disorder (12). Although previous studies have confirmed the association between OSA and MCI, there are significant differences in cognitive impairment profiles between younger patients and those >60 years (13). Younger patients with OSA, who contribute significantly to society and may experience an impact on work performance (14), often remain undiagnosed and untreated. Nevertheless, the identification of younger patients with OSA + MCI continues to be inadequate because of the absence of a practical and convenient screening tool.

Therefore, the purpose of this study was to develop and validate diagnostic models for MCI in OSA patients that are both effective, straightforward and easy to apply across various healthcare settings, even where neurologist expertise is limited. Our models aim to provide OSA patients, especially those who are young or moderately affected, with timely evaluations and interventions that are essential for slowing the progression of cognitive impairment. This proactive approach is vital for reducing the overall disease burden associated with OSA and enhancing patient outcomes.

## 2 Materials and methods

### 2.1 Study design and population

This study was a prospective, observational investigation conducted at the Sleep Medicine Center of the Second Affiliated Hospital of Fujian Medical University, a tertiary hospital in Quanzhou, China, from April 2021 to September 2022. Informed consent was obtained from all participants, and the study was approved by the Institutional Review Board (IRB) of the Second Affiliated Hospital of Fujian Medical University (IRB No. 2021-49).

Young and middle-aged individuals who were newly diagnosed with OSA were enrolled consecutively. The inclusion criteria were: (1) young patients aged 18–44 years or middle-aged patients aged 45–60 years. (2) Underwent overnight polysomnography (PSG) monitoring for at least 4 h. (3) Receiving a diagnosis of OSA. The exclusion criteria were: (1) a prior diagnosis of OSA. (2) Presence of other respiratory diseases or sleep disorders, such as central sleep apnea, obesity hypoventilation syndrome, chronic obstructive pulmonary disease, asthma, insomnia, among others. (4) Previously treated by positive airway pressure machine, oral appliance, or surgery to alter upper airway ventilation.

### 2.2 OSA diagnosis

Participants suspected of OSA underwent overnight PSG in the sleep medicine center. Two types of monitoring devices were utilized in our center: SOMNO screen^™^ plus PSG+ (SOMNOmedics GmbH, Randersacker, Germany) and the Compumedics Grael HD-PSG system (Compumedics, Abbotsford, Victoria, Australia). The monitoring data was manually inspected in accordance with the guidelines outlined in the AASM manual (15) for the purpose of scoring sleep. Participants exhibiting an apnea hypopnea index (AHI) of 5 or more events per hour of sleep, along with symptoms related to OSA, were diagnosed with OSA.

### 2.3 MCI diagnosis

MCI diagnosis was performed prior to PSG monitoring upon patient admission to our sleep medicine center. The MoCA Beijing version^1^ was employed for the assessment of global cognitive function. Cutoff points for diagnosis were established at 14 for individuals with limited literacy, 20 for those with 1 to 6 years of education, and 25 for individuals with 7 or more years of education (16). Scores falling below these cutoff points were classified as MCI. Sensitivity was reported at 83.8%, and specificity was at 82.5%. The assessments were conducted in a quiet interview room at the sleep medicine center by a single experienced physician with expertise in cognitive disorders. Due to the absence of PSG monitoring and other cognitive function assessments, the physician was unaware of PSG parameters and patients’ clinical characteristics.

### 2.4 Other cognitive function assessments

Following the MoCA assessment, subsequent cognitive function evaluations were conducted. The digital span test (DST) was used to assess memory and attention function, with scores recorded for both forward (DST_F) and backward (DST_B) tasks. The total DST score was calculated as the sum of the forward and backward scores.

The Stroop color-word test (SCWT) (version 1935) was used to assess executive function (17). This test comprises three subtasks in which participants were instructed to read three cards in a sequential manner as quickly and accurately as possible. The first card (card A) presents 50 color words in random order (red, yellow, green, and blue) printed in black ink. The second card (card B) features 50 solid color patches in one of the four basic colors. The third card (card C) exhibits 50 color words printed in an incongruent ink color (e.g., the word “red” printed in blue ink). Participants were instructed to identify the ink color of the printed words. The time taken to complete each condition (CWT_A, CWT_B, CWT_C) and the number of correct responses for each condition (NCW_A, NCW_B, NCW_C) were recorded. Two scores were calculated to measure the Stroop interference effect, one based on the reading speed (SIE_T) and the other on accuracy (SIE_N), by using the following formulae: SIE_T = CWT_C − (CWT_A + CWT_B)/2 and SIE_N = NCW_C − (NCW_A + NCW_B)/2. A higher SIE score indicates greater difficulty in the inhibiting interference.

The Chinese-version of the Subjective Cognitive Decline Questionnaire (SCD) (18) was administered to assess subjective cognitive complaints. The total SCD score ranges from 0 to 9, with higher scores indicating better cognitive functioning.

### 2.5 Additional data collection

Age, sex, marital status, year of education, body mass index (BMI) (kg/m^2^), neck circumference (cm), and waist-to-hip (W-H) ratio were collected at the time of PSG monitoring in the evening by clinical nurses. Marital status was categorized into three groups, which were subsequently combined into two categories: with partner and without partner category (reference category). The study gathered PSG parameters from medical records, including AHI (events/h); the distribution of sleep stages N1, N2, and N3 as a ratio of total sleep time; longest durations of apnea and hypopnea; slowest and fastest heart rates; nadir and mean SpO_2_; and sleep efficiency. Excessive daytime sleepiness (EDS) was identified as a significant factor in screening for OSA. The Chinese version of the Epworth Sleepiness Scale (ESS) was utilized to assess daytime sleepiness. The total ESS score ranges from 0 to 24, with higher scores indicating increased levels of sleepiness.

### 2.6 Statistical analysis

Statistical analyses were conducted using SPSS version 26 (IBM Corporation, Armonk, NY, United States) and R version 4.2.1 (R Foundation for Statistical Computing). The normality of continuous variables was assessed using skewness and kurtosis, the graphical method, and the Kolmogorov–Smirnov test. Continuous variables with normal distributions were presented as mean ± standard deviation with the Student’s *t*-test employed for analysis; for non-normally distributed variables, the median (25th, 75th percentiles) was reported, and the Mann–Whitney *U* test was utilized. Categorical variables were expressed as frequencies (percentages), and the chi-squared test was applied. *p* < 0.05 was considered statistically significant.

Enrolled patients were randomly assigned to a training set (70%) and a validation set (30%). The candidate predictors included 20 demographic and clinical characteristics and six cognition factors: age; BMI; sex; year of education; marital status; neck circumference; W-H ratio; AHI; proportions of N1, N2, N3, and REM sleep stages; longest apnea duration; longest hypopnea duration; sleep efficiency; mean SpO_2_; nadir SpO_2_; slowest HR; fastest HR; ESS; DST; DST_F; DST_B; SIE_T; SIE_N; and SCD. These candidate predictors were subjected to univariate regression analyses to determine their association with MCI. Given our study’s limited sample size and exploratory aim, we set a broader threshold for statistical significance to avoid missing key variables in the univariate analysis by using *p* < 0.2 as the criterion. Subsequently, two types of diagnostic models were developed using stepwise multivariable logistic regression analysis. (a) Basic model: utilizing the 20 demographic and clinical characteristic predictors. (b) Cognition factor model: incorporating one significant cognitive factor alongside the demographic and clinical predictors. The fitness of the models was assessed through the Hosmer–Lemeshow (HL) test, while their discriminatory performance was evaluated by calculating the area under the receiver operating characteristic’s curve (AUC). The agreement between predicted probabilities and observed frequencies was examined through the construction of a calibration plot by using the val.prob() function in R. The internal validity of the model was determined by a calibration plot derived from the validation set. Additionally, for clinical application, each model was represented visually with a nomogram. The score for each independent variable was determined from the scale in the first row and added to yield the total points, which was then translated into predicted probabilities in the final row. This study adheres to transparent reporting of a multivariable prediction model for individual prognosis or diagnosis (TRIPOD) guidelines (19, 20).

## 3 Results

### 3.1 Patient and cognitive function characteristics

**Figure 1:**
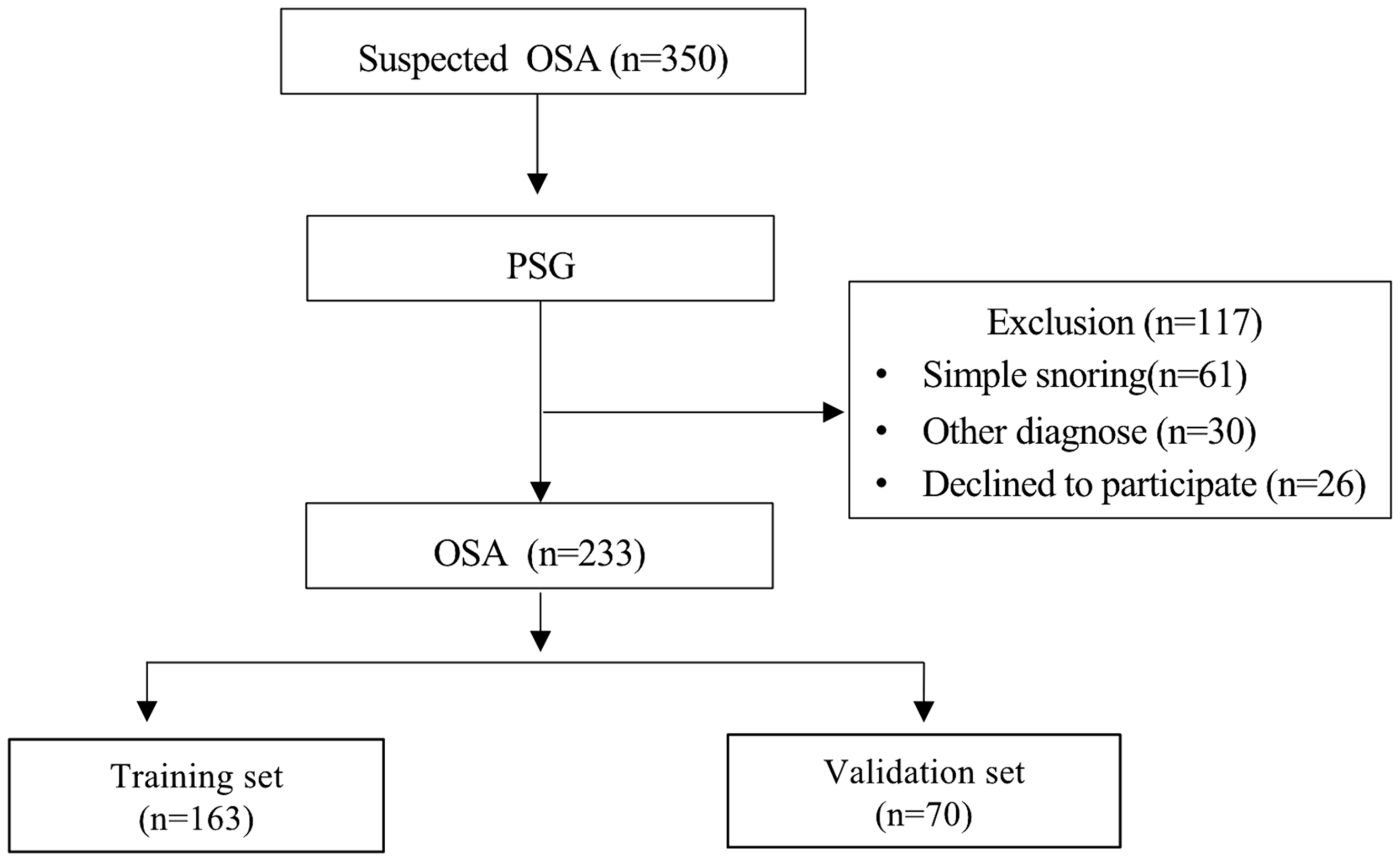
Flowchart of subjects inclusion.

In this study, a total of 350 individuals suspected of OSA underwent PSG monitoring. Of these, 233 patients were diagnosed with OSA and included in the model development (Figure 1), with 89 having MCI and 144 not having MCI. Significant differences were observed between patients with and without MCI across various parameters including age, year of education, BMI, MoCA score, DST-F, DST-B, DST, SIE_T, and SIE_N (*p* < 0.05) (Supplementary Table S1).

### 3.2 Development and validation of MCI-detecting models

#### 3.2.1 Candidate predictors for MCI

Patients with OSA were divided into training and validation sets, comprising 163 and 70 individuals, respectively (Figure 1). With the exception of DST_B and SIE_T scores, patients in the training and validation sets exhibited comparable characteristics (Table 1). Univariate regression analyses identified age, BMI, sex, year of education, longest hypopnea duration, N1 sleep stage ratio, REM sleep stage ratio, mean SpO_2_, DST, DST_F, DST_B, and SIE_T as significant predictors of MCI (*p* < 0.2) (Table 2).

**Table 1:** Univariate analysis of training and validation sets.

Variable	OSA		*t*/*ꭓ*^2^	*p*
	Train data (*n* = 163)	Valid data (*n* = 70)		
Age	38.60 ± 8.45	39.37 ± 9.48	0.615	0.539
Male	141 (86.50)	62 (88.57)	0.187	0.666
Marital status (have partner)	140 (85.89)	54 (77.14)	2.688	0.101
Education	12.96 ± 3.93	12.30 ± 3.78	−1.195	0.233
AHI	41.97 ± 27.40	45.39 ± 26.17	0.887	0.376
BMI	28.10 ± 4.42	28.49 ± 4.35	0.615	0.539
W-H ratio	1.01 ± 0.74	0.96 ± 0.06	−0.599	0.55
Neck_cm	40.74 ± 3.36	40.62 ± 3.75	−0.245	0.807
ESS_score	8.92 ± 4.93	9.34 ± 5.11	0.592	0.555
Longest apnea duration_s	52.26 ± 27.91	55.24 ± 25.64	0.763	0.446
Longest hyponea duration_s	45.91 ± 20.27	51.40 ± 25.66	1.591	0.114
Sleep efficiency	83.52 ± 11.40	83.59 ± 11.83	0.043	0.965
Stage N1	12.50 ± 10.48	12.18 ± 9.82	−0.221	0.826
Stage N2	57.00 ± 14.82	59.53 ± 15.24	1.188	0.236
Stage N3	15.64 ± 11.55	14.34 ± 10.74	−0.804	0.422
Stage REM	12.02 ± 6.19	12.03 ± 6.65	0.008	0.994
Mean SpO_2_	91.01 ± 5.77	90.99 ± 5.75	−0.023	0.982
Lowest SpO_2_	73.31 ± 12.19	73.34 ± 11.10	0.021	0.983
Slowest HR	52.29 ± 6.98	51.93 ± 6.15	−0.38	0.704
Fastest HR	104.00 ± 13.63	106.16 ± 12.75	1.129	0.26
MoCA_optimal score	24.64 ± 3.52	24.39 ± 3.32	−0.511	0.61
MCI_China	56 (34.36)	33 (47.14)	3.392	0.066
DST_F	5.91 ± 1.41	5.83 ± 1.52	−0.414	0.679
DST_B	5.39 ± 1.70	4.80 ± 1.31	−2.847	0.005^**^
DST	11.31 ± 2.73	10.66 ± 2.31	−1.759	0.08
SIE_T	33 (24, 40.25)	28 (21, 37.44)	−2.474	0.013^*^
SIE_N	0 (−1, 0)	0 (−1, 0)	−0.608	0.543
SCD	3.09 ± 2.92	3.74 ± 2.71	1.582	0.115

^*^p < 0.05 and ^**^p < 0.01. AHI, apnea hypopnea index; BMI, body mass index; W-H, waist-to-hip; cm, circumference; ESS, Epworth Sleepiness Scale; s, second; HR, heart rate; MCI, mild cognitive impairment; REM, rapid eye movement; DST, digital span test, the score forward (DST_F) and the score backward (DST_B); SIE_T, time of interference effect; SIE_N, correct number of interference effect; SCD, subjective cognition decline questionnaire.

**Table 2:** Univariate analysis of demographic and clinical characteristics in MCI and non-MCI groups (training set).

Variable	*B*	S.E.	Wald	df	Sig.	Exp. (*B*)	95% CI	
							Lower	Upper
Age	0.035	0.02	3.076	1	0.079^*^	1.035	0.996	1.076
Male	−0.758	0.463	2.677	1	0.102^*^	0.469	0.189	1.162
Marital status (have partner)	0.454	0.507	0.802	1	0.37	1.574	0.583	4.248
Education	−0.272	0.05	29.404	1	<0.001^*^	0.762	0.691	0.841
AHI	0.005	0.006	0.744	1	0.388	1.005	0.993	1.017
BMI	0.108	0.04	7.385	1	0.007^*^	1.114	1.031	1.205
W-H ratio	1.746	2.867	0.371	1	0.543	5.732	0.021	1580.9
Neck_cm	−0.015	0.051	0.085	1	0.771	0.985	0.891	1.089
ESS_score	0.027	0.034	0.67	1	0.413	1.028	0.962	1.098
Longest apnea duration_s	−0.005	0.006	0.75	1	0.387	0.995	0.983	1.007
Longest hyponea duration_s	0.012	0.008	2.286	1	0.131^*^	1.012	0.996	1.029
Sleep efficiency	−0.005	0.014	0.13	1	0.718	0.995	0.967	1.023
Stage N1	0.024	0.016	2.346	1	0.126^*^	1.024	0.993	1.056
Stage N2	−0.002	0.011	0.047	1	0.828	0.998	0.976	1.02
Stage N3	−0.007	0.015	0.248	1	0.619	0.993	0.965	1.021
Stage REM	−0.037	0.027	1.895	1	0.169^*^	0.963	0.913	1.016
Mean SpO_2_	−0.046	0.028	2.708	1	0.1^*^	0.955	0.903	1.009
Lowest SpO_2_	−0.006	0.014	0.203	1	0.653	0.994	0.968	1.021
Slowest HR	0.015	0.025	0.362	1	0.547	1.015	0.967	1.066
Fastest HR	0.005	0.013	0.132	1	0.716	1.005	0.98	1.03
DST_F	−0.482	0.129	13.97	1	<0.001^*^	0.618	0.48	0.795
DST_B	−0.698	0.138	25.506	1	<0.001^*^	0.497	0.379	0.652
DST_total	−0.394	0.078	25.27	1	<0.001^*^	0.674	0.578	0.786
SIE_T	0.034	0.011	10.2	1	0.001^*^	1.035	1.013	1.057
SIE_N	0.004	0.005	0.579	1	0.447	1.004	0.994	1.014
SCD	0.048	0.056	0.721	1	0.396	1.049	0.939	1.172

^*^p < 0.2. AHI, apnea hypopnea index; BMI, body mass index; W-H, waist-to-hip; cm, circumference; ESS, Epworth Sleepiness Scale; s, second; HR, heart rate; MCI, mild cognitive impairment; REM, rapid eye movement; DST, digital span test, the score forward (DST_F) and the score backward (DST_B); SIE_T, time of interference effect; SIE_N, correct number of interference effect; SCD, subjective cognition decline questionnaire.

#### 3.2.2 Basic model

**Figure 2:**
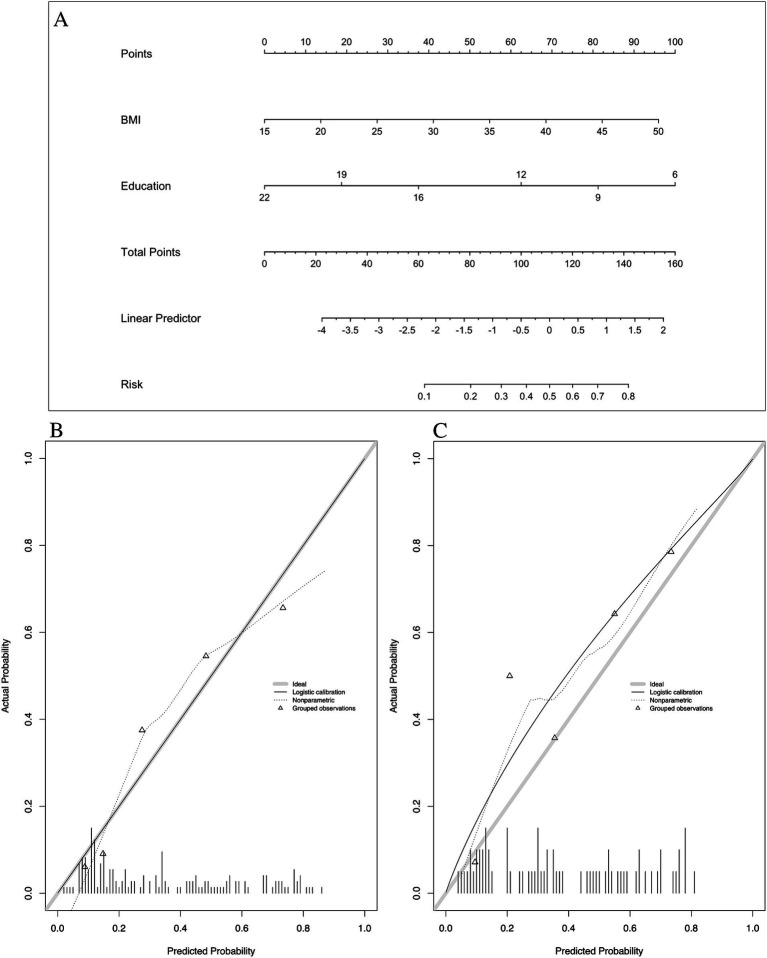
Nomogram and calibration plots of Model 1. **(A)** Nomogram. **(B)** Calibration plots for training set. **(C)** Calibration plots for validation set. BMI, body mass index.

**Figure 3:**
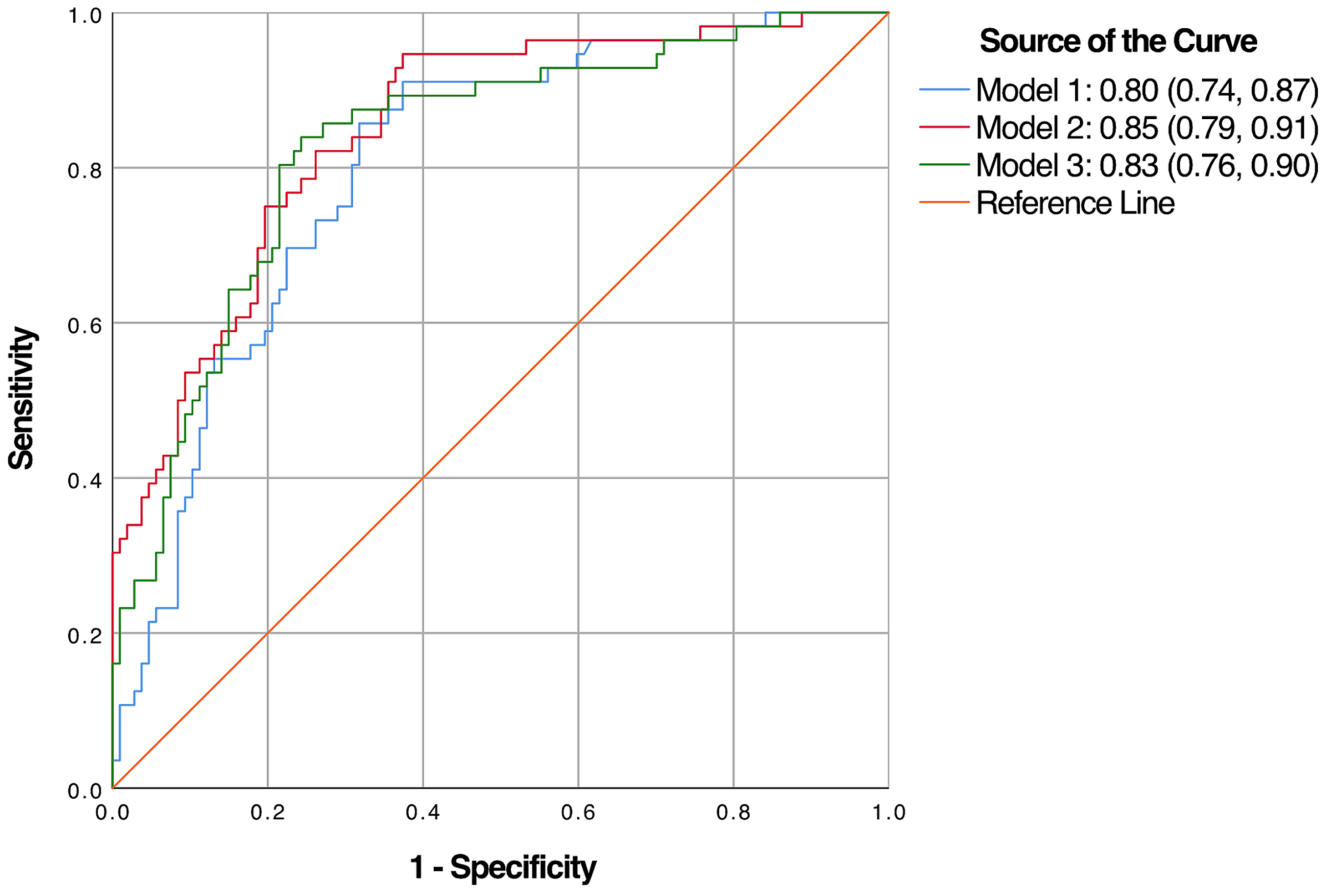
The area under the receiver operating characteristic’s curve (AUC) of the three diagnostic models.

Multivariate analysis, with results reported as odds ratio [95% confidence interval (CI)], identified BMI [1.132 (1.039–1.233)] and years of education [0.754 (0.681–0.835)] as significant predictors, which were subsequently included in the basic model (Model 1) (Table 3). A nomogram for Model 1 was developed (Figure 2A). The model demonstrated good accuracy in detecting MCI, with AUC of 0.803 (95% CI: 0.735–0.872) (Figure 3). The calibration curve for MCI prediction also indicated satisfactory performance (Figure 2B), with the validation set results supporting the nomogram’s predictive accuracy (*p* > 0.05) (Figure 2C).

**Table 3:** Diagnostic models for MCI in patients with OSA.

Model name	Predictor	*B*	S.E.	Wald	Sig.	Exp. (*B*)	95% CI	
							Lower	Upper
Model 1	BMI	0.124	0.044	8.019	0.005	1.132	1.039	1.233
	Education	−0.282	0.052	29.434	<0.001	0.754	0.681	0.835
	Constant	−0.663	1.316	0.254	0.615	0.515	—	—
Model 2	BMI	0.16	0.049	10.567	0.001	1.174	1.066	1.293
	Education	−0.203	0.058	12.24	<0.001	0.816	0.728	0.915
	Stage N1	0.04	0.02	4.139	0.042	1.041	1.001	1.081
	DST_B	−0.594	0.164	13.156	<0.001	0.552	0.4	0.761
	Constant	−0.181	1.427	0.016	0.899	0.834	—	—
Model 3	BMI	0.131	0.045	8.533	0.003	1.14	1.044	1.244
	Education	−0.198	0.058	11.698	0.001	0.821	0.733	0.919
	DST	−0.275	0.093	8.827	0.003	0.759	0.633	0.911
	Constant	1.115	1.451	0.59	0.442	3.049	—	—

BMI, body mass index; DST, digital span test; DST_B, the score of backward of digital span test.

#### 3.2.3 Cognition factor model

**Figure 4:**
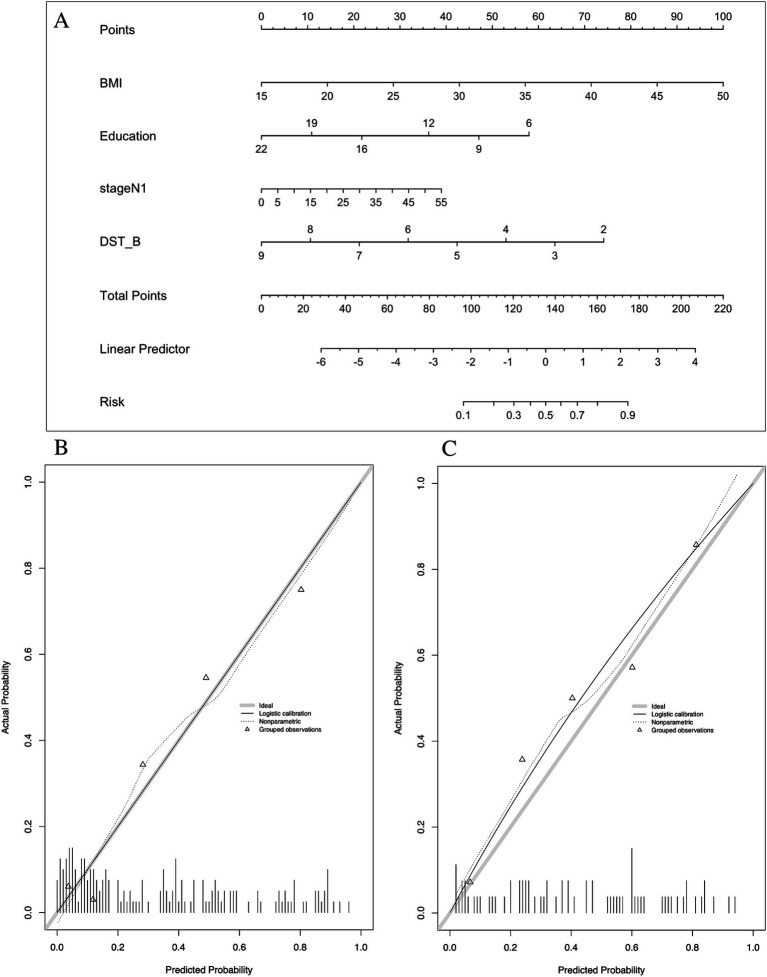
Nomogram and calibration plots of Model 2. **(A)** Nomogram. **(B)** Calibration plots for training set. **(C)** Calibration plots for validation set. BMI, body mass index; DST_B, the score of backward of digital span test.

**Figure 5:**
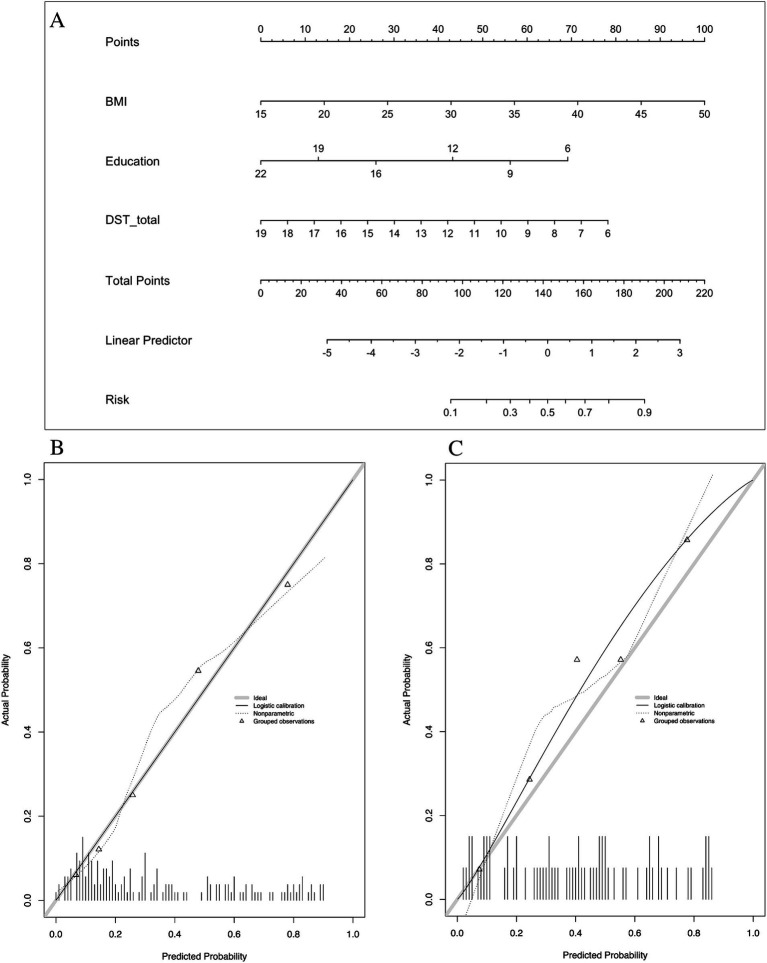
Nomogram and calibration plots of Model 3. **(A)** Nomogram. **(B)** Calibration plots for training set. **(C)** Calibration plots for validation set. BMI, body mass index.

In the development of cognitive factor models, five cognitive factors were sequentially added to the multivariable regression analysis. This process led to the creation of two models: Model 2 and Model 3. Model 2 incorporated BMI [1.174 (1.066–1.293)], years of education [0.816 (0.728–0.915)], N1 sleep stage ratio [1.041 (1.001–1.081)], and DST_B score [0.552 (0.4–0.761)], while Model 3 included BMI [1.14 (1.044–1.244)], years of education [0.821 (0.733–0.919)], and DST score [0.759 (0.633–0.911)]. Both models were statistically significant (Table 3). Corresponding nomograms for each model were constructed (Figures 4A, 5A). The AUCs for Model 2 and Model 3 were 0.849 (95% CI: 0.788–0.91) and 0.83 (95% CI: 0.763–0.896), respectively (Figure 3). Calibration plots for all models showed a good agreement between predicted and observed MCI cases (Figures 4B, 5B). The predictive performance of both nomograms was validated with the validation set demonstrating good predictive accuracy (*p* > 0.05) (Figures 4C, 5C).

## 4 Discussion

The prevalence of MCI was found to be 38.2% among the young and middle-aged population with OSA. Three diagnostic models, each with a corresponding nomogram, were developed. All three models demonstrated strong performance in detecting MCI. The basic model was the most straightforward, utilizing only BMI and years of education. The two additional models incorporating sleep architecture and cognitive factors exhibited improved performance. These models provide a convenient diagnostic tool for healthcare professionals in diverse healthcare settings, enabling prompt and necessary further evaluation and intervention for OSA patients at an increased risk of MCI.

### 4.1 Cognition characteristics

Prior research has predominantly focused on the middle-aged and older patients. In contrast, our study examined a younger cohort with a mean age of 39 years (21, 22). Age plays a crucial role in the development of both OSA and MCI. In middle-aged individuals with OSA, cognitive impairments are predominantly observed in the cognitive domains of executive, attention, and memory. Conversely, older patients showed impairment in global cognition rather than in specific cognitive domains (13). Our results showed significant declines in executive, attention, and memory domains among young and middle-aged patients with OSA + MCI. However, this decline was not reflected in subjective cognitive function reports. The discrepancy between subjective and objective cognitive impairment may lead to reduced emphasis on OSA and lower treatment adherence among patients.

### 4.2 Factors associated with MCI

While year of education has been recognized as a risk factor for MCI, age, sex, and obesity are well-established risk factors for OSA (23), In our study, these factors were also found to be associated with MCI in patients with OSA, and BMI and years of education emerged as independent risk factors for MCI. Higher BMI and fewer years of education were associated with an increased risk of MCI. The literature presents conflicting findings, with some studies reporting a correlation between the severity of OSA, as measured by AHI, and cognitive performance (24, 25), while others found no such association (26). Researchers have put forward that the heterogeneity of OSA complicates the accurate assessment of disease severity using the AHI (12). Conversely, the existing literature lacks sufficient evidence regarding the impact of mild to moderate OSA, as studies have predominantly focused on patients with moderate-to-severe OSA (AHI ≥30) (27). In this study, which included younger patients and those with mild OSA, the association between AHI and cognitive deficits was not observed. Despite being a hallmark symptom of OSA, EDS was observed in only 41.4% of male and 44.6% female patients (28). Therefore, AHI and ESS score may not be the most reliable predictors for MCI in patients with OSA.

Although the precise mechanisms linking OSA to cognitive decline remain a subject of debate (24, 29, 30), several key factors such as sleep fragmentation, chronic intermittent hypoxia, oxidative stress, and cerebrovascular alterations have been identified as potential contributors to the pathophysiology of cognitive deficits in OSA patients (24, 29). However, consensus on the specific pathways through which these deficits manifest is lacking. It is also possible that these factors interact in complex ways, indicating the need for further investigations to elucidate their interplay in the development of OSA-associated cognitive impairment. In our study, only stage N1 was incorporated into Model 2, suggesting a potential association between an increase in light sleep and MCI. This finding aligns with previous research conducted on older patients (age, >65 years) (25). Increased light sleep may impair cognition through two potential mechanisms. First, it could degrade nighttime sleep quality, leading to diminished daytime attention (30). Second, it may reduce deep sleep duration, resulting in decreased cortical activation and the structural deterioration of gray and white matter in key brain regions such as the frontal and parietal cortices, temporal lobes, hippocampus, and cerebellum, thereby adversely impacting cognitive function (29).

### 4.3 Comparison of models

Based on literature review, there is a scarcity of convenient models to detect MCI in patients with OSA. Prior studies have primarily focused on exploring peripheral body fluid biomarkers (e.g., plasma, serum, urine) and neuroimaging biomarkers (utilizing MRI-or PET-based) linked to MCI. One model utilized urinary AD7c-NTP in conjunction with other selected factors to evaluate the cognitive risk in OSA patients (10). Although the model demonstrated a high level of accuracy in assessing cognitive risk (AUC: 0.841), concerns remain regarding the practicality of using urinary AD7c-NTP as a predictor, particularly in terms of financial burden and convenience. In our study, we developed three straightforward models for MCI in OSA patients. Our developed models, as well as the urinary biomarker models, displayed comparable performance in predicting MCI. Another model for predicting MCI was established at the time of writing this report (11). However, that model was originally designed based on data from middle-aged and older individuals, with one predictive factor derived from a self-compiled lifestyle scale. Although the specific profiles of cognitive deficits in OSA patients is an ongoing debate, consensus has been reached on deficits in attention, memory, and executive function (31, 32). Including additional cognitive domains in diagnostic models may enhance their predictive accuracy. Specifically, the assessment of memory and attention using DST was found to be particularly effective for predicting MCI in OSA patients. This finding is consistent with previous imaging biomarker studies for MCI, which emphasizes the importance of the basal nucleus and cortical nucleus subregions in predicting the MoCA score (33). These brain regions are integral to memory and attention functions, which can be evaluated through DST.

In the context of discriminatory performance, an AUC >0.7 is generally considered acceptable, and one >0.8 is particularly good. Our models which achieved this level of discriminatory performance without the incorporation of physiological predictors have demonstrated a satisfying performance. The MoCA typically requires around 10 min to complete (9), whereas our study observed that the DST can be efficiently completed within 3–5 min. The DST’s streamlined design, with its two tasks and clear instructions, may also reduce the evaluator bias, often associated with the more complex MoCA. The simplicity of this approach could enhance the DST’s utility as an accessible screening tool for non-specialists and may improve the consistent identification of MCI, especially in settings where complex assessment protocols remain impractical. Furthermore, a brevity test might improve patient compliance and reduce the risk of assessment discontinuation, which is crucial for ensuring that MCI cases are not overlooked.

### 4.4 Models’ application

The basic model was the most straightforward, requiring only BMI and years of education. It enables the prediction of MCI presence without the need for PSG monitoring or other cognitive function assessments. Consequently, this model may serve as a valuable screening tool in large-scale populations. Model 2 required an additional factor related to sleep architecture, determined by PSG monitoring. This model exhibited the most discriminative performance. Compared to the basic model, the remaining two models each incorporated a cognitive function variable, which require a quiet environment for the assessment of cognitive functions to be accurate. Hence, the basic model is the most accessible, suitable for use in various settings, particularly in busy outpatient clinics and primary healthcare facilities. Model 2 is an appropriate choice when the PSG report is accessible.

### 4.5 Strengths and limitations

Considering the elevated risk of MCI in patients with OSA and the potential impact of CPAP treatment on cognitive deterioration (7, 34), identifying MCI in these patients is crucial. The simplistic yet efficient models we devised can be customized for various contexts. This study has concentrated on young and middle-aged subjects, thereby contributing novel evidence from a relatively younger demographic. The guidance provided by interviewers during cognitive assessments may significantly influence participants’ performance. Therefore, all cognitive evaluations in this study were conducted by a single physician specialized in cognitive disorders. This approach likely minimized biases stemming from inconsistencies in intra-and inter-rater reliability.

This study has some limitations. First, the study cohort was solely based on outpatient clinic samples from a single institution. This may limit the generalizability of our models. Although the clinic and community samples shared similar cognitive profiles, subtle differences in power of attention and quality of episodic memory were observed between them (35). Thus, it is imperative to validate the models’ performance by using community-based samples and data from multiple centers. Second, despite enrolling all eligible patients during the study period, the sample size was smaller than the optimal size. The validity of our developed models should be confirmed by future studies with larger sample sizes.

### 4.6 Further research

Early detection of MCI may facilitate the prediction of potential impairment of decision-making capabilities (36). This predictive information can guide healthcare providers in offering additional support to patients when making decisions about CPAP treatment. Informing patients about their risk of MCI may help them make informed choices and improve adherence to CPAP therapy, potentially leading to modified long-term management for OSA. Timely early intervention is crucial for mitigating the risk of cognitive decline associated with aging and OSA. Further research is required to investigate the potential impact of clinical application of MCI nomograms on CPAP adherence and the prevalence of MCI. To enhance the accessibility of these models and minimize the need for human resources, efforts should be directed towards developing cognitive function assessments that are web-based or utilize virtual reality technology. Given the feasibility of incorporating DST assessments into mobile applications, this approach may prove to be more accessible than other complex assessments.

## 5 Conclusion

We developed three straightforward diagnostic models for MCI in patients with OSA, predicated on a minimal set of easily accessible parameters, namely BMI, education years, DST performance, and N1 sleep duration. These models serve as a practical diagnostic instrument for healthcare providers in diverse settings, streamlining the process of identifying OSA patients at an increased risk of MCI and requiring further assessment and intervention. Incorporating these models into routine clinical use can contribute to mitigate the progression of cognitive impairment in OSA patients, leading to more effective management of this common disorder. The models’ reliance on straightforward and accessible measures enhances their clinical utility and potential for widespread application.

## Data Availability

The original contributions presented in the study are included in the article/Supplementary material, further inquiries can be directed to the corresponding author.

## Footnotes

^1^www.mocatest.org
