# Supplementary material for: Development of a diagnostic model for detecting mild cognitive impairment in young and middle-aged patients with obstructive sleep apnea: a prospective observational study
Source: Front Neurol. 2024 Aug 21;15:1431127. doi: 10.3389/fneur.2024.1431127 (PMC11371584; doi:10.3389/fneur.2024.1431127)
Supplement: Supplementary file 1 [file Table_1.DOCX]

Supplementary Table 1 Demographic and clinical characteristics of MCI and non-MCI groups.

| Variable | OSA | | t/ꭓ2 | *P* |
| --- | --- | --- | --- | --- |
|  | non-MCI(n=144) | MCI(n=89) |  |  |
| Age | 37.58±8.58 | 40.85±8.71 | -2.81 | 0.005** |
| Male | 73(82) | 130(90.3) | 3.34 | 0.07 |
| Marital status  (have partner) | 76(85.4) | 118(81.9) | 0.47 | 0.493 |
| Education | 14.19±3.58 | 10.45±3.19 | 8.08 | <0.001** |
| AHI | 41.28±25.78 | 45.77±28.86 | -1.23 | 0.219 |
| BMI | 27.58±3.95 | 29.25±4.88 | -2.73 | 0.007** |
| W-H rito | 0.96±0.06 | 1.06±0.99 | -1 | 0.32 |
| Neck_cm | 40.80±3.26 | 40.56±3.81 | 0.5 | 0.619 |
| ESS_score | 9.06±5.00 | 9.02±4.98 | 0.06 | 0.952 |
| Longest apnea duration_s | 53.94±27.84 | 51.89±26.31 | 0.56 | 0.579 |
| Longest hyponea duration_s | 45.16±17.18 | 51.44±28.02 | -1.91 | 0.059 |
| Sleep efficiency | 83.97±11.04 | 82.85±12.26 | 0.72 | 0.473 |
| Stage N1 | 11.44±9.64 | 13.96±11.09 | -1.83 | 0.068 |
| Stage N2 | 57.87±15.09 | 57.58±14.84 | 0.14 | 0.889 |
| Stage N3 | 16.00±12.04 | 14.04±9.95 | 1.28 | 0.202 |
| Stage REM | 12.16±6.31 | 11.81±6.36 | 0.41 | 0.683 |
| Mean S_P_O_2_ | 91.58±4.70 | 90.07±7.06 | 1.79 | 0.075 |
| Lowest S_P_O_2_ | 73.85±11.21 | 72.46±12.83 | 0.84 | 0.402 |
| Slowest HR | 51.72±7.49 | 52.93±5.22 | -1.34 | 0.183 |
| Fastest HR | 104.67±14.40 | 104.61±11.62 | 0.04 | 0.971 |
| MoCA | 26.66±1.81 | 21.17±2.69 | 17.03 | <0.001** |
| DST_F | 6.27±1.35 | 5.27±1.38 | 5.46 | <0.001** |
| DST_B | 5.78±1.53 | 4.29±1.30 | 7.92 | <0.001** |
| DST | 12.06±2.38 | 9.58±2.26 | 7.88 | <0.001** |
| SIE_T | 25(19.25,33) | 36(28,47.5) | -6.017 | <0.001** |
| SIE_N | 0(-1,0) | 0(-1.54,0) | -1.697 | <0.001** |
| SCD | 3.12±2.97 | 3.54±2.70 | -1.1 | 0.275 |
| * p<0.05 ** p<0.01; Definition of abbreviations: MCI = mild cognitive impairment; AHI = apnea hypopnea index; BMI = body mass index; W-H = waist-to-hip; cm = circumference; ESS = Epworth sleepiness scale; s = second; HR = heart rate; MoCA= optimal Montreal Cognitive Assessment score; REM= Rapid Eye Movement; DST = digital span test, the score forward (DST_F) and the score backward (DST_B); SIE_T = time of interference effect; SIE_N = correct number of interference effect; SCD = subjective cognition decline questionnaire. | | | | |
